# Influencing Factors and Differences in Born Aggregometry in Specialized Hemostaseological Centers: Results of a Multicenter Laboratory Comparison

**DOI:** 10.1055/a-1827-7025

**Published:** 2022-08-22

**Authors:** Thorsten Kaiser, Karin Liebscher, Ute Scholz, Christian Pfrepper, Jeffrey Netto, Tim Drogies, Oliver Tiebel, Ralf Knöfler, Michael Krause

**Affiliations:** 1 Institute of Laboratory Medicine, Clinical Chemistry and Molecular Diagnostics, University of Leipzig, Paul-List-Str., Leipzig, Germany; 2 Institute of Transfusion Medicine and Clinical Hemostaseology, Klinikum St. Georg gGmbH, Delitzscher Straße, Leipzig, Germany; 3 MVZ Laboratory Reising-Ackermann MD and Colleagues, Strümpellstraße, Leipzig, Germany; 4 Division of Hemostaseology, University Hospital Leipzig, Liebigstraße, Leipzig, Germany; 5 Medical Central Laboratory Altenburg, Am Waldessaum, Altenburg, Germany; 6 Institute of Clinical Chemistry and Laboratory Medicine, Medical Faculty of Technical University, Dresden, Germany; 7 Department of Paediatric Haemostaseology, University Hospital Carl Gustav Carus, Dresden, Germany

**Keywords:** inherited/acquired platelet disorders, platelet physiology, platelet immunology, platelet glycoproteins

## Abstract

**Introduction** Light transmission aggregometry (LTA) is regarded as the gold standard in platelet function diagnostics. However, there is a relevant degree of interlaboratory variability in practical applications.

**Objective** The aim of the present study was to develop a practicable laboratory comparison on LTA and to analyze differences and influencing factors in regard to standardization in five specialized hemostaseological centers.

**Methods** The study was performed on 30 patients in total. Each center performed LTA on blood samples from six healthy volunteers (three men and three women) using the inductors collagen (Col), adenosine diphosphate (ADP), arachidonic acid (ARA), and ristocetin. The LTA was performed three times using different methods as follows: (1) International Society on Thrombosis and Haemostasis recommendations with identical reagents, (2) in-house protocols and the identical reagents; and (3) in-house protocols and in-house reagents.

**Results** A total of 396 measurements of 30 probands were performed. Even after standardization of the protocol and using identical reagents, there were significant differences between the centers regarding the final and maximum aggregation ( *p* = 0.002 and <0.001) and further significant differences in the maximum and final aggregation according to the wavelength of the device used to measure the LTA (PAP-8: 430 nm, APACT 4004: 740 nm [ *p* < 0.001 each]). Using identical reagents but individual inductor concentrations and laboratory protocols also resulted in different maximum and final aggregation. The largest differences were seen with Col and ristocetin; there were significant influences from the reagents' manufacturers in the results of aggregometry for the inductor Col ( *p* < 0.01) but not for ADP, ARA, and ristocetin.

**Conclusion** In this study, we proved that there are significant influences from the used aggregometers, inductors concentrations, and manufacturers. These results illustrate the challenges and importance of standardization of LTA.

## Introduction

Light transmission aggregometry (LTA), which was developed independently by Born and O'Brien, 1 2 3 4 is currently the gold standard in platelet function diagnostics. Different recommendations by national and international professional societies are available. 5 6 7 In addition, the reagents used for diagnosis (inductors) and their concentrations exhibit a high degree of interlaboratory variability in practical applications. 8 The aim of the present study was to investigate differences and influencing factors to harmonize the diagnostic laboratory methods of the LTA in specialized hemostaseological laboratories in central Germany.

## Materials and Methods

### Study Design

A multicenter study of thrombocyte function tests using the Born-based LTA 1 was performed in five coagulation centers as follows: (1) University Hospital Leipzig, Institute for Laboratory Medicine, Clinical Chemistry and Molecular Diagnostics; (2) University Hospital Dresden, Institute for Clinical Chemistry and Laboratory Medicine; (3) Klinikum St. Georg gGmbH, Institute for Transfusion Medicine and Clinical Haemostaseology; (4) Medical Central Laboratory Altenburg; and (5) MVZ Labor Leipzig Dr. Reising-Ackermann and Colleagues. The study was performed on 30 patients in total. Each center performed LTA on blood samples from six healthy volunteers (three men and three women). The LTA was performed with the following four inductors: (1) adenosine diphosphate (ADP), (2) arachidonic acid (ARA), (3) collagen (Col), and (4) ristocetin. Three different protocols (methods) in a fixed order were used. All examination steps, including preanalytics and the final qualitative (subjective) evaluation, were strictly defined and documented. This was followed by a peer-review procedure within the group.

In method A, standardized reagents were used in the International Society on Thrombosis and Haemostasis (ISTH) standard 7 measurement protocol which had been sent centrally in advance and produced ready for use in the respective centers. Method B included the performance of the LTA with the centrally dispatched reagents according to the in-house LTA measurement protocol previously used in the respective center. In method C, the LTA was finally performed with the in-house reagents according to the in-house LTA measurement protocol ( Table 1 ).

**Table 1:** Description of the methods for LTA

	Inductor concentrations in accordance with	LTA-protocol in accordance with
Method A a	ISTH standard	ISTH standard
Method B a	ISTH standard	In-house
Method C b	In-house	In-house

Abbreviations: ISTH, International Society on Thrombosis and Haemostasis; LTA, light transmission aggregometry.

a Identical inductor reagents were used in all centers ( Table 3 ).

b In-house protocol and reagents were used.

### Test Patients

The inclusion criteria for the 30 patients were as follows: objectively healthy, men-to-women gender ratio of 50% each, age >18 years (minimum: 26 years and maximum: 57 years), platelet counts between 150 and 450 Gpt/L, nicotine withdrawal >30 minutes, and time difference since intake of fatty meals and caffeine-containing drinks >2 hours. Exclusion criteria were the intake of medication or food supplements within the previous 10 days prior to blood collection which might influence platelet function (including hormonal contraception), as well as known platelet dysfunctions.

### Preanalytics

The samples were collected by intravenous blood collection according to the following criteria (analogous to ISTH standard): venous stasis as short as possible (maximum 1 minute) and direct puncture with 21 G puncture needle without extension tube. The blood collection system used was a tube with 0.106 mol/L (3.2%) trisodium citrate solution (Sarstedt, Germany). None of the samples collected were transported via pneumatic tube systems. Hemolysis was not detected in any samples.

Performance of the LTA took place within 4 hours of blood collection, observing a pause of at least 30 minutes between transport and the start of platelet processing (start of centrifugation).

After determination of the platelet count in the blood sample using an automatic blood count system, 10 mL of citrated whole blood was centrifuged at 200 g for 10 minutes at room temperature (21°C) to produce platelet-rich plasma (PRP). After taking the PRP and determining the platelet count (>100 Gpt/L), the sample was centrifuged again at 1,500 g for 15 minutes to obtain low platelet plasma (PPP). The platelet count of the PRP was not adjusted to a certain platelet value with PPP (17). Immediately before the start of the measurements, the required final concentrations of the different reagents were prepared on site in the participating laboratory.

### Reagents and Devices

The four inductor reagents—ADP, ARA, Col, and ristocetin—were used for the measurements according to the ISTH standard (method A) and the respective in-house LTA (method B; Table 2 ).

**Table 2:** Inductor reagents with the respective manufacturers for methods A and B

Inductors	Manufacturer
ADP	Fa. Probe & Go (Lemgo, Germany)
Arachidonic acid	Fa. Probe & Go (Lemgo, Germany)
Collagen (horse tendon)	Fa. Probe & Go (Lemgo, Germany)
Ristocetin	Fa. Mölab (Langenfeld, Germany)

The inductor reagents were ordered centrally (same batch) and shipped in lyophilized form to the participating centers at 2 to 8°C according to the manufacturer's specifications. Immediately before the start of the measurements, the required final concentrations were prepared on site at the participating institute. The following inductor concentrations were used for the measurements according to ISTH standard (method A) and the respective in-house LTA (methods B and C), whereby the laboratory's own inductor reagents (method C) are also listed ( Table 3 ).

**Table 3:** Inductor final concentrations and manufacturers for methods A to C

Inductors	According to ISTH standard	In-house (IH) labor 1	IH labor 2	IH labor 3	IH labor 4	IH labor 5
Collagen (µg/mL)	2	190 µg/mL ^c^	10 µg/mL ^b^	8 µg/mLd	8 µg/mLa	190 µg/mL ^c^
Arachidonic acid (mmol/L)	1	1 mmol/L ^b^	1.64 mmol/L ^b^	1.64 mmol/L ^b^	1.64 mmol/L ^a^	1.64 mmol/L ^b^
ADP (µmol/L)	2	2; 5; 20 µmol/L ^c^	0.5; 2.5; 5 µmol/L ^b^	0.6;1.25; 5 µmol/L ^d^	5 µmol/L ^a^	1; 2 µmol/L ^c^
Ristocetin (mg/mL)	1.2	1.2 mg/mL ^b^	0.5; 1.5 mg/mL ^b^	0.4; 0.75; 1.5 mg/mL ^b^	0.375; 0.75; 1.5 mg/mLa	0.3; 0.6; 1.5 mg/mL ^b^
Manufacturer: ADP: ^a^ HART Biologicals/Haemochrom, ^b^ Mascia Brunelli, ^c^ Mölab, and ^d^ Biopool Stago. Arachidonic acid: ^a^ HART Biologicals / Haemochrom, and ^b^ Mölab. Collagen: ^a^ HART Biologicals/Haemochrom, ^b^ Mölab, ^c^ Mölab (calf skin), and ^d^ Probe & Go (Horm). Ristocetin: ^a^ HART Biologicals/Haemochrom and ^b^ Mölab.						

Abbreviations: ADP, adenosine diphosphate; ISTH, International Society on Thrombosis and Haemostasis.

### Measurements

After transferring the PRP into a cuvette, the platelet aggregation was measured at 37°C using the Born-based LTA. 1 This method photometrically determines the turbidity of the plasma at a defined wavelength with the aid of light transmission and records the aggregation of the platelets as a decrease in turbidity over time in a curve. The PPP of the patients is regarded as the reference point for 100% transmission. By using the turbid PRP, the maximum light transmission is absorbed and thus considered as 0% transmission. After the addition of the inductors of platelet aggregation (ADP, ARA, Col, or ristocetin), the aggregation of the platelets in the sample begins and causes a decrease in turbidity and, thus, an increase in light transmission. 7 Table 4 shows the instruments used by each participating laboratory.

**Table 4:** Specifications of the laboratory equipment used by the participating laboratories

	Site 1	Site 2	Site 3	Site 4	Site 5
Manufacturer	Mölab	APACT	APACT	APACT	Mölab
Instrument	PAP-8	4004	4004	4004	PAP-8
Wavelength (nm)	430	740	740	740	430

The maximum and the final aggregation in percentage after 10 minutes for all inductors were used for the evaluation. A final aggregation >60% are considered to be within the reference range. A peer review procedure for the optical evaluation of the aggregation curves was performed anonymously. The evaluation of basic pathological platelet aggregation, as well as the presence of a possible disaggregation in the ADP-induced aggregation curve were recorded. By consensus, disaggregation was defined as >15% after 10 minutes.

The raw data of all sites' results are available in the Supplementary Material S1.

### Statistics

Data were analyzed using Excel 14.0 (Microsoft Corporation, Redmond, Washington, United States) and SPSS 23.0 (IBM, Armonk, New York, United States). A *p* -value of <0.05 was considered statistically significant. Due to the lack of normal distribution of the results (Shapiro–Wilk test *p* < 0.001), nonparametric tests were used for analysis. Where appropriate, the Wilcoxon's signed-rank or the Mann–Whitney *U* -test was used to assess whether the mean ranks differ across the categories of methods, cases, and sites.

## Results

A total of 396 measurements of 30 patients were performed (five sites; six patients per site; different numbers due to additional ADP concentrations of the in-house protocol: sites 1, 2, 5; 84 and site 3, 4: 72 measurements, see Table 3 for details).

### Comparison of the Results of Method A between the Different Laboratories

First, the results of method A (identical protocol and reagents for all sites) were compared between the different laboratories. The final and maximum aggregation was significantly different ( *p* = 0.002 and <0.001). The median results of the sites ranged from 71.5 to 90.0% maximum aggregation and 69.0 to 88.5% final aggregation.

As in method A, the reagents, as well as the protocols did not differ between the sites; a possible explanation for this could be the use of different aggregometers using different detection wavelengths (PAP-8 was used by sites 1 and 5 with a wavelength of 430 nm and APACT 4004 was used by sites 2, 3, and 4 with a wavelength of 740 nm [ *p* < 0.001 each]). The analysis revealed significant differences in the maximum and final aggregation according to the devices ( Table 5 ).

**Table 5:** Differences in the maximum and final aggregation of all inductors based on the devices and wavelengths used

Wavelength (nm)		Maximum aggregation (%)	Final aggregation (%)
430	Median ( *n* )	77.5 (48)	74.5 (48)
740	Median ( *n* )	89 (72)	87.2 (72)
Total	Median ( *n* )	87 (120)	85.2 (120)

The differences concerned the inductors ADP, Col, and ristocetin ( *p* = A: 0.035 and 0.019; C: 0.039 and 0.022; and R: 0.039 and 0.2). For ADP, there was only a nonsignificant difference ( *p* = 0.172 and 0.172), most likely due to three outliers in site 4. After elimination of site 4 from this analysis, ADP was also significant ( *p* = 0.007 each).

### Impact of the Individual Laboratory Protocol on Light Transmission Aggregometry (Methods A versus B)

Using identical reagents but individual laboratory protocols (methods A vs. B) also resulted in different maximum and final results. Detailed analysis of different inductors revealed significantly higher results using a Col (horse tendon) concentration of 10 µg/mL in the in-house protocol compared with 2 µg/mL, the recommended ISTH concentration (site 2, median maximum = 95.0 vs. 86.6% and a final aggregation = 95.0 vs. 86.6%, *p* = 0.004 each).

A significant difference was found when comparing the ristocetin inductor concentration of 1.5 mg/mL (sites 2, 3, 4, and 5) with the ISTH recommendation of 1.2 mg/L (median of the maximum and final aggregation = 89.7 vs. 85.2 and 85.5 vs. 77.3%, respectively, *p* = 0.043 and 0.037).

Despite the different inductor concentrations (1 vs. 1.64 and 1.67 mM), the results for the inductor ARA were not significantly different.

Interestingly, there were also no significant differences for ADP with regard to the maximum and final aggregation despite the different inductor concentrations (1, 2, 2.5, and 5 µM).

### Influence of Reagent Manufacturers on Aggregometry Results (Methods B vs. C)

There were no significant influences from the reagents' manufacturers on the results of aggregometry for the inductors ADP, ARA, and ristocetin.

However, the Col (horse tendon) used showed an influence on the results of the aggregation. At identical concentrations, there were significant differences at sites 2 and 4 for the maximum and final aggregation ( Table 6 ; site 2: maximum aggregation, *p* = 0.009; final aggregation, *p* = 0.009; site 4: *p* = 0.004 and 0.002, respectively). It turned out that the level of aggregation, although horse tendon was used in both sites, depended on the manufacturer. The highest results were present in the horse tendon of Hämochrome, followed by Probe & Go, and finally Mölab.

**Table 6:** Influence of collagen manufacturers on aggregometry results

Collagen (method)		Maximum aggregation (%)	Final aggregation (%)
Site 2			
10 µg/mL Horse Tendon, Probe & Go (B)	Median Minimum Maximum	95 89 96	94 89 96
10 µg/mL Horse Tendon, Mölab (B)	Median Minimum Maximum	88.5 84.5 91	88.5 84.5 91
Site 4			
8 µg/mL Horse Tendon, Probe & Go (B)	Median Minimum Maximum	90 88.4 93	88 84.7 89.8
8 µg/mL Horse Tendon, HART Biologicals/Haemochrom (C)	Median Minimum Maximum	94 92.7 95.3	92.4 91 94

Note: Although horse tendon collagen was used in sites 2 and 4, results were significantly different. *p* -Values: site 2: maximum aggregation *p* = 0.009, final aggregation *p* = 0.009 between Probe & Go and Mölab; site 4: *p* = 0.004 and *p* = 0.002 between Probe & Go and HART Biologicals/Haemochrom, respectively.

Interestingly, using horse tendon Col 2 µg/mL (Probe & Go) and calfskin Col 190 µg/mL (Mölab), there were no significant differences with respect to maximum and final aggregation.

### Detailed Results of Patients with Aggregations below 60%

In 27 measurements of nine patients, the maximum aggregation of at least one measurement was below 60%. ADP was the most common inductor in these cases (ADP, *n* = 19; ARA, *n* = 3; ristocetin, *n* = 3; and Col, *n* = 2).

Disaggregation occurred in 19 of the 396 measurements. There were eight disaggregations after stimulation with ristocetin and 11 after ADP stimulation with the following inductor concentrations: 7 × 2.0 µM, 1 × 2.5 µM, and 3 × 5.0 µM.

No disaggregation was detected when stimulated with Col or ARA.

The detailed results of all patients with at least one aggregation below 60% are displayed in Table 7 .

**Table 7:** LTA results of all patients with at least one maximum aggregation <60%

Inductor	Method	Conc. Site 2	P9 (%)	P10 (%)	Conc. Site 4	P20 (%)	P21 (%)	P22 (%)	P23 (%)	Conc. Site 5	P26 (%)	P28 (%)	P29 (%)
ARA	A	1 mM	95	91	1 mM	86	91	10	91	1 mM	58	100	68
	B	1.64 mM	90	93	1.64 mM	89	90	91	89	1.64 mM	59	108	63
	C	1.64 mM	84	92	1.64 mM	90	90	94	90	1.64 mM	64	95	75
COL	A	2 µg/mL	85	94	2 µg/mL	83	89	91	88	2 µg/mL	55	98	68
	B	10 µg/mL	94	95	8 µg/mL	89	88	93	91	190 µg/mL b	64	101	62
	C	10 µg/mL	87	91	8 µg/mL	93	95	95	93	190 µg/mL b	58	88	71
ADP	A	2 µM	84	40 a	2 µM	38 a	46 a	61 a	45 a	2 µM	81	52 a	69
	B	2.5 µM	88	34 a	5 µM	79	88	79	57 a	1 µM	47	79	59
	C	2.5 µM	31	55	5 µM	59 a	89	84	51 a	1 µM	46	78	56
	B	5 µM	95	56						2 µM	53	84	70
	C	5 µM	90	94						2 µM	51	77 a	58
RISTO	A	1.2 mg/mL	89	88	1.2 mg/mL	91	90	85	91	1.2 mg/mL	59	95	63
	B	1.5 mg/mL	91	90	1.5 mg/mL	90	93	96	96	1.5 mg/mL	54 a	98	68
	C	1.5 mg/mL	89	91	1.5 mg/mL	91	97	89	97	1.5 mg/mL	59	100	80

ARA, arachidonic acid; COL, collagen; Conc., concentration of inductors for corresponding sites; LTA, light transmission aggregometry; M, method (A = ISTH [International Society on Thrombosis and Haemostasis], B = in-house protocol with identical inductor manufacturer, C = in-house protocol with in-house reagents); P, proband; RISTO, ristocetin.

a Disaggregation >15%.

b Calf skin.

## Discussion

The results obtained from five established coagulation laboratories in central Germany show that despite several national and international guidelines 8 9 10 11 on LTA, the method continues to be handled differently. The differences concern preanalytical conditions, the type and manufacturer of the reagents used and their final concentrations, as well as the aggregometers which differ in the wavelengths of the detection systems. The interlaboratory variability has been confirmed in several studies. 12 13 14

The aim of this study was to develop a practicable laboratory comparison and to systematically analyze differences and relevant influencing factors on LTA in five specialized hemostaseological centers to achieve standardization.

Exact specifications for preanalytics and the reagents were defined during the planning of the study within the framework of a consensus procedure. This took place after critical discussion of the recommendations of the ISTH, 8 the Association of the Scientific Medical Societies in Germany (AWMF), 15 and further published data. 7 9 10 11 15 16 17

The integration of the three described study arms, which in all cases are processed in parallel, allows a differentiated assessment with regard to the influence of the different protocols, as well as the reagents normally used at the sites and their critical evaluation in comparison to the ISTH standard.

The use of batch-identical reagents distributed to the participants in lyophilized form, with production of the ready-to-use reagents immediately prior to the performance of the tests, ensures the exclusion of preanalytical differences on the reagent side.

For method A which was based on the ISTH standard (with the exception of ADP), significantly different results were found between the two device systems. The missing discrepancy for ADP was probably caused by the three probands of site 4 with extremely low results described below. After elimination of these outliers from statistical analysis, the results also significantly differed between the wavelengths ( *p* = 0.012). The median results for maximum, as well as final aggregation were higher for the 740-nm wavelength (red light) compared with 430 nm (blue light). Without violating the respective reference ranges, these results indicate the influence of the two different detection wavelengths on the diagnostic results. According to our literature research, there is little data on the influence of wavelength.

Hayward et al pointed out the influence of the aggregometer used and criticized the insufficient information from the manufacturers on important technical details of the devices and their missing but necessary standardization. 19 A discussion on the influence of different wavelengths on aggregometric measurements was found in a dissertation published in 2012 20 in which the results of LTA were compared with the aggregometers PAP-4 (697 nm) and PAP-8 (430 nm). In contrast to our results, however, higher maximum aggregations were found when using the lower wavelength (430 nm). In this publication, the influence of wavelength is discussed and the physical principles of light scattering are pointed out. According to this, platelets with a diameter of 1 to 4 μm correspond approximately to the wavelength of the light source, and the so-called Mie scattering occurs during light transmission through the nonactivated PRP. After addition of the agonist and the associated activation, the platelets change shape and aggregates are formed that are significantly larger than the wavelength used. As a result, the light is increasingly scattered.

When comparing method A (ISTH standard) with method B (identical reagents with local protocol) at one site (740 nm system), significantly higher maximum aggregation results were found with Col which can be explained by the higher final concentration of the inductor (horse tendon, 10 µg/mL) in comparison to the ISTH standard in method A. At two other sites, also with 740 nm device systems, the protocol is based on a Col concentration of 8 µg/mL (horse tendon) from different reagent manufacturers. However, in this case, no significant differences were found compared with the ISTH standard. At the two sites with a 430-nm device system, no significant difference was detected between the 190 µg/mL calfskin Col with 2 µg/mL horse tendon Col and an equivalent platelet aggregation strength can be speculated.

For the Col obtained from horse tendon, significant differences between different manufacturers occurred in two laboratories using identical concentrations and devices (APACT 4004) for both final and maximum aggregation. Compared with Probe & Go, the aggregation results were higher for the Hemochrome reagent and lower for Mölab. Consequently, it could be assumed that the degree of aggregation was manufacturer-dependent: Hemochrome > Probe & Go > Mölab.

The considerable influence of Col from different species and manufacturers can be assumed as known and is confirmed by the results. Even after thorough literature research, however, no final details can be assured. Farndale and Siljander point out that in a manufacturing process based on a non-proteolytic cleavage of the tendon material, the addition of bioactive material, such as decorin, cannot be ruled out. 21 Decorin interacts with Col fibrils and Guidetti et al could demonstrate a direct influence on platelet reactivity. 22

From the group of investigations on ARA-stimulated aggregation, one case will be specifically discussed that underlines the importance of orientation to the final concentration recommended in the ISTH standard. The final concentration of 1-mM ARA used in method A according to the ISTH standard revealed pathological results. The parallel investigation of the material in method B with a final concentration of 1.64-mM ARA, however, led to a regular course of aggregation. In this specific case (P22), it can be assumed that the test patient did not report acetylsalicylic acid (ASS) or non-steroidal anti-inflammatory drugs (NSAID) consumption or that there was a functional disorder in the sense of an ASS-like defect. 22 In this case, however, even an analytical error cannot be completely excluded, as no material for remeasurement was available. This is an indication that the recommended ISTH concentration for ARA was chosen sensibly, as slight aggregation disorders can thus be detected well.

## Limitations

In principle, the use in this study of locally recruited test patients for practical considerations at the sites must be pointed out. In this respect, interindividual influences on the results cannot be ruled out and may have significant impact on the results of the sites. In spite of the inclusion criteria for test patients to be “objectively healthy,” a mild platelet dysfunction may have been present. Accordingly, three test patients from site 4 who showed reduced aggregations after induction with ADP are conspicuous and two of these (P20, P21) were siblings. The results of site 4 were confirmed in later additional blood samples. These findings reveal a general limitation of this study. Since we want to achieve a practicable laboratory comparison, the number of test patients per site was limited to six and a pathological control was not available. Furthermore, the aspect of reproducibility of results as intraday and interday variance was not considered in this study, besides the coefficient of variation compared with other clinical chemistry parameters is assumed to be higher. This should be the subject of further studies.

## Conclusion

In conclusion, our protocol allows to carry out a laboratory comparison which sensitizes the participants to individual differences in terms of the Born-based LTA. The results obtained at five specialized diagnostic centers illustrate the importance of the highest possible standardization to obtain comparable findings. The final concentrations of the inductors should be based on the recommendations of the ISTH to minimize the risk of a lack of detection of mild platelet dysfunctions.

In addition, the establishment of method-specific reference values must be underlined, as this is the only way to exclude the influence of physical effects, such as different wavelengths, in the instrument systems used on the findings. In the context of Col -induced aggregation, it must be assumed that the reagents of different suppliers will have different effects on platelets depending on the manufacturing process.

Our results underline the importance of the current AWMF guidelines for platelet function diagnostics which were last updated in 2018. LTA deals clearly with other special methods of functional assessment. As one of the last areas of hemostaseological diagnostics, the establishment of an interlaboratory test regime must also be established for platelet function diagnostics to control adequate and maximally standardized diagnostics with regard to comparability of the results across the diagnostic laboratories.

It is planned to carry out this laboratory comparison among the participants on a regular basis and, if applicable, to expand it to achieve more comparable results for patient diagnostics in the long term.
